# Long Non-Coding RNA HOTAIR Promotes Human Osteosarcoma Proliferation, Migration through Activation of the Wnt/b-Catenin Signaling Pathway

**DOI:** 10.1155/2023/9667920

**Published:** 2023-02-07

**Authors:** Hang Li, Shijian Fu, Wenbo Wang

**Affiliations:** Orthopedics Department, The First Affiliated Hospital of Harbin Medical University, Harbin 150081, Heilongjiang, China

## Abstract

LncRNA HOTAIR exhibited different effects in human cancers. However, the role of HOTAIR was not reported in osteosarcoma. This study aimed to explore the function of HOTAIR in osteosarcoma. Firstly, we examined HOTAIR expression in breast cancer tissues by the RT-qPCR assay and examined HOTAIR protein expression via immunocytochemistry, to chemical assay, and Western blot. Then, for further exploring the function of HOTAIR, we also examined it by CCK-8 and transwell assays. Downregulation of HOTAIR was detected in osteosarcoma, which predicted poor prognosis of patients with osteosarcoma. Moreover, cell migration, invasion, and proliferation were suppressed by HOTAIR overexpression in osteosarcoma. Furthermore, LPR5 was a direct target of HOTAIR, which was upregulated in osteosarcoma. Especially, the upregulation of LPR5 could impair the suppressive effect of HOTAIR in breast cancer. HOTAIR was found to negatively regulate the EMT and Wnt/*β*-cadherin pathways in osteosarcoma. HOTAIR repressed the progression of osteosarcoma via regulating LPR5 and suppressing the Wnt/*β*-cadherin pathway. Our findings will provide a positive reference for studying the function of HOTAIR in osteosarcoma.

## 1. Introduction

Osteosarcoma (OS) is caused by bone cells' abnormal differentiation and proliferation. According to epidemiological data, the incidence of OS is around 0.2–3/100000 per year [1]. In children and teenagers with a high rate of malignancy, it is the most prominent primary bone tumor. OS usually shows a high tendency to metastatic spread [2]. OS is also associated with procedures that may require them: chemotherapy and radiology [3, 4]. However, in recent years, the survival rate of patients with OS accompanied by distant metastases has not been significantly improved [5, 6], the effect of chemotherapy has not been significantly improved, and the treatment of OS is still controversial. Therefore, new therapeutic targets need to be found to provide clinical treatment options to improve the survival rate.

Long noncoding RNAs (LncRNAs) are a family of 200 nt length RNA molecules [7, 8]. While proteins cannot be encoded, lncRNAs can participate in multiple levels of gene expression regulation, including epigenetic, transcriptional, and post-transcriptional modification, thus taking part in a variety of in vivo pathophysiological processes, including cancer proliferation and invasion [9, 10]. Gene expression analysis suggests LncRNA HOTAIR is situated at human chromosome 12q13 and consists of 5 short exons and 1 long exon within the antisense strand of the HOXC gene cluster [11]. HOTAIR is a typical molecular occurrence of epigenetic malignancy that has been shown to have great importance in the production and prediction of different tumors. New research suggests that HOTAIR is a chromatin regulation system that routinely controls tumor metabolism, proliferation, etc. [12–14]. A number of studies indicate that HOTAIR could bind these genes to the repressive polycomb complex 2 (PRC2) directly and silently [11]. In addition, HOTAIR may also interact with the LSD1/REST complex and H3K4 histone demethylation. HOTAIR knock-out can prevent invasion and metastasis of the breast tumor. The production of HOTAIR by improving the epithelial-mesenchymal transformation in gastric cancer was shown to facilitate cellular invasion and migration. HOTAIR is reported to be involved in Wnt/*β*-catenin signaling through directly decreasing HIF-1 expression.

Therefore, in this study, we selected the human osteosarcoma cell line SAOS-2 and the human osteoblast cell line OB3. We applied RT-qPCR Western blot to our cells. In addition, we analyzed the lncRNA HOTAIR expression and its interaction with LPR5, attempting to establish a connection between HOTAIR and LPR5 in OS. Our findings indicate that HOTAIR and LRP5 expressions are upregulated, and levels of LRP5 expression have a positive correlation with HOTAIR in OS tissues and cell lines.

## 2. Methods

### 2.1. Cell Lines and Culture

Human osteosarcoma cell line SAOS-2 and human osteoblast cell line OB3 were purchased from the ScienCell Research Laboratories and the American Type Culture Collection (ATCC) (Manassas, VA, USA).

### 2.2. RT-qPCR

The TRIzol reagent was used to extract complete RNA (Sangon Biotech, Shanghai, China). For the qRT-PCR experiment, 2 *μ*g of complete RNA for reverse transcript and cDNA synthesis is used with TransScript ® II Reverse Transcriptase (TransGen Biotech Co,, Beijing, China). Quantitative, real-time PCR experiments have been carried out with PerfectStartTM Green qPCR SuperMix (TransGen Biotech Co., Beijing, China). The results were normalized to GAPDH, a gene with constitutive expression. The PCR primers are for HOTAIR forward, 5′-GGTAGAAAAAGCAACCACGAAGC-3′; for HOTAIR reverse, 5′-ACATAAACCTCTGTCTGTGAGTGCC-3′; and for GAPDH, 5′-TGTTCGTCATGGGTGTGAA-3′ (forward) and 5′-ATGGCATGGACTGTGGTCAT-3′ (reverse). Using the 2-Ct process, the relative MFI2 and FOXP4 expression levels were identified and normalized.

### 2.3. Western Blot

A complete protein was extracted for 30 minutes, and the protein concentration was measured using a BCA protein measuring kit (Thermo Scientific, MA, USA). The Mammalian Complete Protein Extraction Kit (TransGen Biotech Co., Beijing, China) was used on ice. The corresponding numbers of total proteins, which were transferred to 0.22 *μ*m polyvinylidene fluoride (PVDF) membranes (Millipore, Billerica, MA, USA.) and incubated with LRP5 (1 : 1000) or GAPDH (1 : 1500) anticorps are 12 per cent SDS-polyacrylamide gel electrophoresis (SDS-PAGE). The manufacturer's (Beyotime) identification of enhanced chemiluminescence (CEL) and the quantified densitometry of the band amplitude (Quantity One software; Bio-Rad, Hercules, CA) as proteins measured.

### 2.4. siRNA Transfection

As previously defined, the pENTR-shHOTAIR vector was constructed (Liu et al., 2014). In brief, Genepharmacy Technology (China) synthesized unique oligonucleotides targeting HOTAIR: sense, 5′-GATCCGCCACATGAACGCCCAGAGATTTTCAAGAGAAATCTCTGGGCGTTCATGTGGTT TTTTG-3′; antisense, 5′-AATTCAAAAAACCACATGAACGCCCAGAGATTTCTCTTGAAAATCTCTGGGCGTTCATGTGGC G-3′. The pENTR-shHOTAIR plasmids and empty vectors were then transfected into U2OS cells, and G418 (400 *μ*g/ml) was chosen for the HOTAIR overexpression subclones. To validate the upregulation of HOTAIR, real-time PCR was conducted.

### 2.5. Cell Viability Assay

The manufacturers' instructions using a cell counting kit-8 (CCK-8) (Dojindo, Kumamoto, Japan) observed the cell viability of siRNA Duplex osteomarcoma cells at 24 h, 48 h, and 72 h. Briefly, 1 to 104 cells were placed on 96-well tissue culture plate for 24, 48, and 72 hours. OS cells were subsequently treated with CCK-8 for 1 hour at 37°C. OS cells were used to measure 450 nm of absorption with a microplate reader, Thermo Plate (Rayto Life and Analytical Research, Co., Ltd.).

### 2.6. MTT Assay

The MTT test determined the cell proliferation effect of HOTAIR. In short, 2–10^3^ cells/button were sown on 96-well plates and cultivated periodically. At the specified timepoints, the 10 mg MTT solution (5 mg/ml; Sigma-Aldrich) was used for each well, and the reaction was completed with 200 *μ*l DMSO 2 hours later. The absorbance on a microplate reader was estimated at 570 nm. The experiment was repeated atleast three times.

### 2.7. Transwell Assay

The researchers used 24-well transwells with 8 *μ*m pores (Corning Costar, Inc., Corning, NY, USA) and conducted migration and invasion assays, respectively. In the migration assay, the upper transwell chamber was filled with a noncoated membrane with 2–10^4^ OS cells suspended in a 100 *μ*l serum-free culture medium. The upper chamber for the invasion procedure was replaced with 3 to 10^4^ OS cells plated without FBS in 100 *μ*l of the required culture medium. In the samples, 500 *μ*l culture medium containing 20 percent FBS was found in the lower culture chamber. In a wet climate, the cells were cultivated for 24 hours at 37°C and 5% CO_2_. Set to 100 percent methanol for 30 minutes, 0.5% violet (Sigma, St. Louis, MO, USA) cells have been dyed over a 20-minute span, and a phase-contrast microscope (Olympus, Tokyo, China) has been compared.

## 3. Result

### 3.1. LncRNA HOTAIR Expression was Increased in OS Tissue

**Figure 1:**
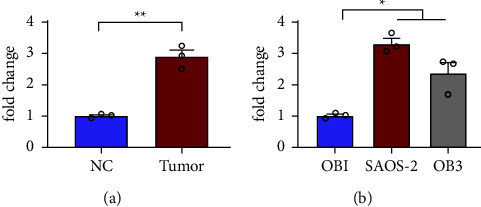
HOTAIR expression was increased in OS tissues. ^*∗*^*P* < 0.05, ^*∗∗*^*P* < 0.01.

Figure 1 shows that HOTAIR expression was increased in OS tissues.  Figure 1(a) shows the expression of HOTAIR in the tissues of OS.  Figure 1(b) shows that high HOTAIR expression was detected in OS cell lines. First, in OS tissue and normal tissue using RT-qPCR, lncRNA expression was studied. It was shown that lncRNA HOTAIR expression was higher than in normal tissue. Similarly, high HOTAIR expression was also observed in the OB-3 and SAOS-2 tumor cell lines.

### 3.2. HOTAIR Downregulation Prevented OS Cell Proliferation

**Figure 2:**
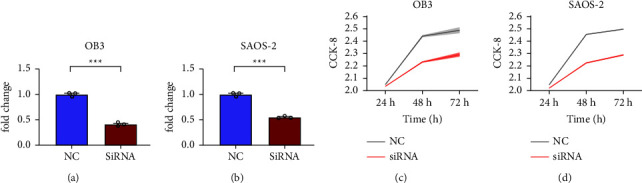
Downregulation of HOTAIR inhibited the progression of OS. *P*  < 0.05, ^*∗∗*^*P*  < 0.01, and ^*∗∗∗*^*P* < 0.001.

The knockdown experiment was conducted to demonstrate the effect of HOTAIR on the proliferation of OS cells. Figures 2(a) and 2(b) show the HOTAIR expression in normal OB-3, SAOS-2 cell lines, and siRNA knockdown cell lines, and the expression of HOTAIR in OS cell lines is successfully knocked down. The cell proliferation was measured in the cell lines OB-3 and SAOS-2 by the CCK-8 assay. As shown in Figures 2(c) and 2(d), in the CCK-8 assay, we observed that in two OS cell lines, declining HOTAIR expression substantially suppressed cell proliferation. A central role of HOTAIR in the proliferation of OS was indicated by the findings.

### 3.3. HOTAIR Downregulation Inhibited the Invasion and Migration of OS Cells

**Figure 3:**
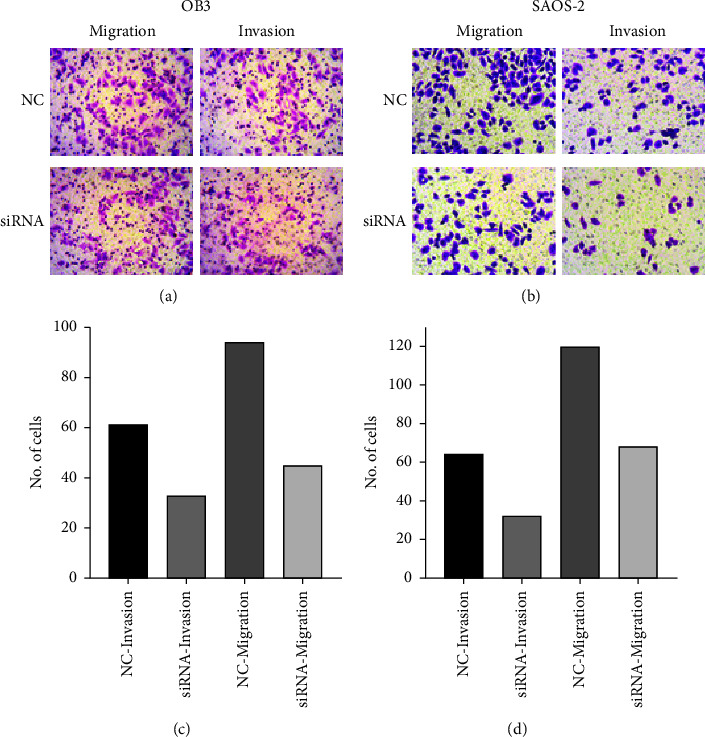
Downregulation of HOTAIR has prevented OS cell migration and invasion.

Figures 3(a) and 3(b) show the representative images of the cell invasion results of HOTAIR knockdown on OB-3 and SAOS-2 cells. It was used to compare cell invasiveness before and after HOTAIR knockdown by transwell assay. Figures 3(c) and 3(d) show the quantification of outcomes for OS cell lines in cell invasion and migration assays. As shown in Figures 3(c) and 3(d), the downregulation of HOTAIR inhibited the migration of OS cell lines OB3 and SAOS-2 in the transwell migration assay.

### 3.4. Protein LPR5 was Positively Correlated with HOTAIR Expression

**Figure 4:**
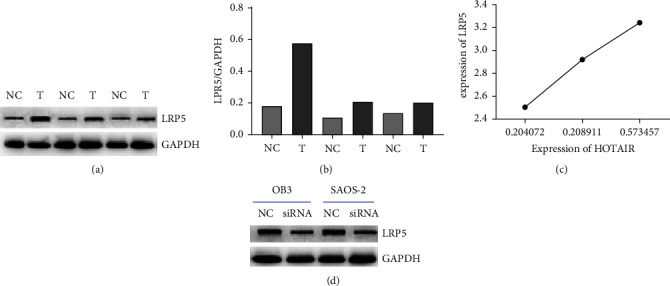
LPR5 was upregulated in osteosarcoma tissues.

LRP5, which is a part of the LDL receptor family, comprises the VLDL receptor and the apolipoprotein E receptor 2. LRP5, which is a coreceptor of Wnt, is situated between Frizzled and Kremen receptors on the osteoblast membrane. Figure 4(a) shows the protein expression of LRP5 in osteosarcoma tissues detected by WB. It has been demonstrated that LPR5 is a potential oncogenic protein in OS. Figure 4(b) shows the quantification of the results for LPR5 expression in osteosarcoma tissues (^*∗*^*P* < 0.05). To explore whether there is any relationship between the expression of LPR5 and HOTAIR, we examined the correlation between HOTAIR and LPR5 expression. Figure 4(c) shows that the expression of HOTAIR and LPR5 has a positive correlation. As shown in Figure 4(c), we found a positive correlation between the two expressions. HOTAIR knockdown reduced the expression of LPR5 (Figure 4(d)). It suggested that the possible mechanism by which HOTAIR promotes cancer growth is the regulation of LPR5 expression.

## 4. Discussion

A number of lncRNAs have played a significant part in essential cellular processes in previous research, for example, gene expression regulation, and post-transcriptional modification [10, 15]. LncRNA expression is increasingly known to be implicated in pathological advancements such as the growth of human cancer, culminating in unchecked proliferation [10, 16]. An increased understanding of the biological role of lncRNA can also provide new approaches for human OS diagnosis and treatment. OS has been the most common bone cancer in the younger crowd, like children and young adults. It has remained poorly treated and has a poor prognosis [17]. Wnt is the secreted protein that causes tumor growth and skeletal development [18]. It includes 10 Frizzled receptors and 19 Wnt ligands, as well as LRP5, LRP6, and two low-density lipoprotein receptor-related protein (LRP) coreceptors [19]. As Wnt ligands were connecting to LRP5/6, the carboxyl end of Lrp5/6 was phosphorylated, and a binding position for Axin was formed [20]. It leads to the b-catenin level increased in the cytoplasm and nucleus [21, 22]. Ultimately, this leads to abnormal gene expression and tumor formation. It has been demonstrated that the expression of LRP5 is a common event in OS [23].

Despite numerous studies, the molecular mechanisms of OS proliferation and metastasis have remained unclear [24]. With the deepening understanding of lncRNA, it has been recognized that lncRNA is involved in every aspect of human physiology and pathology, which may be related to the molecular mechanism of proliferation and metastasis of OS. Earlier research found HOTAIR participates in multiple tumor forming and metastasis pathways [25]. In esophageal squamous cell cancer, HOTAIR knockdown may reduce the ability of cells to proliferate, migrate, and invade the extracellular matrix [26]. In gastric cancer, HOTAIR expression is upregulated to promote the proliferation of gastric cancer cells [27]. In human OS tissues, HOTAIR expression was substantially upregulated. The role of HOTAIR in the incidence and invasion of OS has been explored in this report.

Our findings found that HOTAIR downregulation prevented OS cell proliferation. CCK-8 and transwell assay findings indicated that HOTAIR knockdown by RNA interference greatly decreased cell proliferation, migration, and invasion in OS cells. To sum up, we demonstrated that HOTAIR and LRP5 expressions were upregulated, and levels of LRP5 expression were positively correlated with HOTAIRs in OS tissues and cell lines. However, there are still some limitations in our study, such as limited data. In the future, we need to collect more data for more in-depth data analysis, which will greatly improve the reliability and scientificness of our results.

## Data Availability

The datasets used and/or analyzed during the current study are available from the corresponding author on reasonable request.
